# Comparison of patient satisfaction with pharmaceutical services of postal pharmacy and community pharmacy

**DOI:** 10.4102/hsag.v24i0.1105

**Published:** 2019-07-30

**Authors:** Nishern Govender, Fatima Suleman

**Affiliations:** 1 Discipline of Pharmaceutical Sciences, University of KwaZulu-Natal, Durban, South Africa

**Keywords:** pharmacy, postal, community, patient satisfaction, South Africa, survey

## Abstract

**Background:**

The growing drive in South Africa to contain medicine cost has seen the emergence of postal pharmacy as an alternative mechanism to dispense chronic medicines. Patient satisfaction with pharmaceutical services has received limited attention in South Africa.

**Aim:**

The aim of this study was to compare the level of patient satisfaction with pharmaceutical services between postal and community pharmacies.

**Setting:**

The research was conducted in the eThekwini Municipality, KwaZulu- Natal Province, South Africa in July and August 2014.

**Methods:**

A cross-sectional quantitative study using a randomised, telephonic questionnaire survey was conducted. Selected land telephone numbers were called until a sample size of 250 community pharmacy participants and 125 postal pharmacy participants was obtained.

**Results:**

Nine hundred and five telephone calls were made to obtain a sampling frame of 375 (41.44%) respondents, 250 for community and 125 for postal. After adjusting overall satisfaction by removing financial satisfaction, there was no significant difference between satisfaction in the two groups (*p* = 0.471). Postal pharmacy participants reported a higher level of financial satisfaction (*p* = 0.001). Community pharmacy participants reported a higher level of satisfaction with counselling or explanation (*p* = 0.028) and less medicine wastage (*p* < 0.001).

**Conclusions:**

Patient satisfaction with pharmaceutical services provided by either community or postal pharmacy was not significantly different. However, community pharmacies tend to address patients’ specific concerns more effectively. With the move to National Health Insurance, policymakers need to ensure that they provide high-quality pharmaceutical services and are more inclusive of community pharmacies to deliver quality care.

## Introduction

Patient satisfaction has become an increasingly popular indicator of healthcare systems and services. Patients’ personal evaluation of care has become the most common method of assessing its quality, viability and sustainability (Larson, Rovers & MacKeigan 2002; MacKeigan & Larson 1989). The provision of pharmaceutical services has not been excluded from this evaluation, as they have become a central component of healthcare systems (Johnson et al. 1999; Panvelkar, Saini & Armour 2009).

Pharmaceutical care is a professional practice and involves the responsible provision of pharmacotherapy to achieve positive healthcare outcomes (Hepler & Strand 1990; Traverso et al. 2007). The provision of pharmaceutical services has significant benefits to patients. It can result in improvements in communication, convenience and courtesy and can lead to increased use and ultimately improved healthcare outcomes (Kassim, Collins & Berkowitz 2012; Volume et al. 2001).

Several studies have demonstrated the association between patient satisfaction and health outcome (Hall et al. 1990; Kane, Maciejewski & Finch 1997; McCombs et al. 1995). Patient satisfaction with pharmaceutical care can impact patients’ health and their health-related quality of life. It has also been associated with positive health-related behaviours, such as improved adherence and more effective use of healthcare resources (Johnson et al. 1999; McCombs et al. 1995).

The practice of pharmacy in South Africa is regulated by an independent statutory body, the South African Pharmacy Council (SAPC), which mandates the provision of quality pharmaceutical services in accordance with standards relating to Good Pharmacy Practice (GPP) (South African Pharmacy Council 2010). Good Pharmacy Practice describes the dispensing procedure to be followed by pharmacists and divides this process into three phases: interpreting and evaluating the prescription, preparing and labelling the prescribed medicine and providing information and instructions to the patient to ensure the safe and effective use of the medicine (SAPC 2010).

The SAPC allows for dispensing pharmacies to be registered under two categories – a community pharmacy (independent, retail or corporate) or an institutional pharmacy (located at private or public institutions). A third category of service provision that recently became available in South Africa is a postal pharmacy, which requires patients to collect medicines from a post office. However, in the absence of a separate category, the SAPC requires them to be registered as a community pharmacy and to conform to the relevant rules and regulations (SAPC 2010). To provide counselling and information services to patients, these postal pharmacies are making use of telephonic communication via call centres.

Medication for chronic diseases in South Africa has traditionally been provided by community pharmacies (Gilbert 1998). Over the last two decades, increased medical coverage, burden of disease and expenditure on chronic medicines have seen the emergence of postal pharmacies. For the purposes of this study, postal pharmacy will also refer to mail or courier pharmacies.

The recent expansion of the postal pharmacy is primarily driven as a mechanism by private healthcare funders and pharmacy benefit managers (PBMs) to control chronic medicine costs (Rupp 2013). Their growth has occurred despite continuing questions about the claimed cost advantages of this type of medication service (Kirking, Ascione & Richards 1990; Rupp 2013). Several studies conducted in the United States have shown that overall medicine costs are higher for postal pharmacies (Carroll 2014; Carroll et al. 2005; Johnsrud, Lawson & Shepherd 2007; Khandelwal et al. 2012). In a study conducted by Carroll et al. (2005), researchers concluded that postal pharmacy was less expensive to a patient because of decreased co-payments but was more expensive for a funder. More recently, Carroll (2014) reported that both private and public healthcare funders pay more for prescriptions dispensed at a postal pharmacy.

The South African public healthcare sector has not been immune to these developments in pharmaceutical care models, with the introduction of the integrated chronic model in August 2013 (Naidoo 2013). In October 2013, the National Department of Health issued a tender notification for the Provision of an Alternative Chronic Medication Access programme for the public sector, which was implemented in February 2014 (Department of Health 2013).

Beyond the purported cost advantages of using postal pharmacy, there have been anecdotal concerns expressed by patients and community pharmacists around their use. These include the patient’s freedom to choose a pharmacy service and the legality of healthcare funders implementing high co-payments to incentivise patients to use designated service providers (Medical Chronicle 2012, 2015; Power 2015). In addition, more specific concerns have been voiced with respect to product integrity, medicine wastages, convenience and prescription delivery mechanisms (Magubane 2014; Medical Chronicle 2012). Kirking et al. (1990) and Rupp (2013) described similar concerns with patients using postal pharmacies in the United States.

Although considered an important determinant of health outcomes, patient satisfaction within the pharmacy context has only received attention to a limited degree (Panvelkar et al. 2009). Patient satisfaction with healthcare has been researched in different settings and among special populations, such as those with disabilities or chronic diseases (Xiao & Barber 2008). In 1989, researchers in the United States developed and validated a multidimensional instrument to measure patient satisfaction with pharmacy services (MacKeigan & Larson 1989), which was further validated in 1994 and updated in 2002 to include pharmaceutical care (Larson et al. 2002). A 2009 review of international literature found 24 articles in which the main focus was measuring patient satisfaction in community pharmacies (Panvelkar et al. 2009).

There is limited research exploring patient satisfaction with different pharmacy settings in South Africa. Much of the current research comparing community and postal pharmacies is concerned with the cost benefit of these settings, with most comparisons being done in high-income countries (Clark, Siracuse & Garis 2009; Valluri et al. 2007).

Birtcher and Shepherd (1992) surveyed university employees in the United States who used a postal pharmacy and reported that 88% did so because of the cost savings achieved. Smith and Coons (1993) also measured satisfaction with postal and community pharmacies in the United States, and although there was no direct comparison between the two types, consumers of postal pharmacy services reported a greater level of satisfaction.

Research conducted by Johnson et al. (1997) compared the level of satisfaction between postal and traditional pharmacy services among enrolees of a managed care organisation in Arizona and Southern California. The authors reported that patrons of the mail service pharmacy appeared to have a higher level of satisfaction, but that the difference was not large and may not be of practical significance.

There is no research in South Africa comparing the perceptions of patients with regard to the service they receive from postal and community pharmacies. As this method of service delivery is growing in South Africa, it is important to assess patient satisfaction to inform policymakers about their perceived successes and challenges. Thus, the aim of this study was to compare the level of patient satisfaction with pharmaceutical services between postal and community pharmacies.

## Materials and methods

A cross-sectional quantitative study was conducted during July and August 2014 to establish the satisfaction of patients who access their chronic medicines using community and postal pharmacies. A telephonic survey was adapted from the Satisfaction with Pharmacy Services Questionnaire (MacKeigan & Larson 1989) and a survey developed by Johnson et al. (1997) as these questionnaires had been developed previously and validated in 4 separate studies. Internal-consistency reliability coefficients for the scales exceeded 0.50. The survey was carried out on people who had fixed telephone landlines in the eThekwini Municipality, by means of random selection of telephone numbers.

The eThekwini municipality is located on the east coast of KwaZulu-Natal Province (Co-operative Governance and Traditional Affairs 2011). It covers an area of 2292 km² and is populated by almost 3.5 million people, which accounts for 34% of the province’s population (Statistics South Africa 2011). The majority of the population in the province is black ethnic group (74%) with Indians as the next largest race (17%). Furthermore, the province houses the third largest number of people covered by medical aid schemes at approximately 1.3 million, which is 14.9% of the total number of beneficiaries in South Africa (Council for Medical Schemes 2015). The eThekweni telephone directory (which is divided into three regions, that is North Coast and surrounding areas, South Coast and surrounding areas, and Durban and surrounding areas) consists of approximately 144 000 household landline contacts (Statistics South Africa 2011, 2014).

A sample size calculator (see http://www.raosoft.com) was used to calculate the sample size with a 5% margin of error and 95% confidence interval. The sample size was divided into two categories – patients using community pharmacy and patients using postal pharmacy. Based on current patronage of postal pharmacy (Council for Medical Schemes 2015), respondents using postal pharmacy were oversampled (in a 1:2 ratio) to ensure adequate representation in the final sample.

Random telephone numbers were generated using Microsoft Excel (version 2010) and filtered for only telephone numbers containing area codes within the eThekwini Municipality (Telkom SA 2014). The selected landline telephone numbers were called between 08:00 and 21:00 (to allow for people working either during the day or at night to be included in the sample) during July and August 2014, until a sample size of 250 community pharmacy respondents and 125 postal pharmacy respondents was obtained.

To be eligible for selection, a participant must have had a chronic prescription dispensed by either a community pharmacy or a postal pharmacy in the previous 30 days and must have been 18 years or older.

Responses were statements of opinion ranging from strongly disagree to strongly agree, with a score of 1 defined as strongly disagree and 5 defined as strongly agree, such that a higher score indicated a higher level of satisfaction. Scores for questions where a higher score would indicate a lower level of satisfaction were transposed, that is, 1 became 5, 2 became 4, 3 remained 3, 4 became 2 and 5 became 1, to ensure that a higher score indicated a higher level of satisfaction. The questionnaires covered demographic information and satisfaction with pharmaceutical services, which in this study is the dispensing process as defined by GPP (South African Pharmacy Council 2010) and was assessed in four categories: general satisfaction, satisfaction with financial aspects, satisfaction with technical competence and satisfaction with counselling or explanation (which was defined as face-to-face communication for community pharmacy and telephonic communication for postal pharmacy). Category scores were calculated by summing the individual question scores in each category, dividing the sum by the number of questions in the category and multiplying that by 10. The resulting score was reported to a maximum of 10, with a higher score indicating a higher level of satisfaction for the respective category. Questions addressing specific concerns were adjusted to result in Yes or No answers by equating the responses on a satisfaction score to either ≤ 3 or ≥ 4, respectively, for negatively worded questions and the opposite for positively worded questions.

Data were coded and entered into Microsoft Excel (version 2010), and community and postal pharmacy respondents were compared using Student’s *t*-test for continuous variables and chi square tests for independence for nominal variables using Minitab version 16, as well as McNemar tests to determine if there are differences on a dichotomous dependent variable between two related groups. Chi square can be employed to test the difference between two or more actual samples (Key 1997). Rejection happened on the 5% level of significance.

### Ethical considerations

Ethics approval for the study was obtained from the Humanities and Social Science Research Ethics Committee of the University of KwaZulu-Natal (HSS/0154/013). Informed consent was obtained from participants prior to the start of the interview.

## Results and findings

A total of 905 telephone calls were made to obtain a sampling frame of 375 subjects, which constituted a 41.44% response rate. Those participants that did not respond to the survey indicated that they were not interested in participating in the survey (*n* = 206) or that they did not take any chronic medication (*n* = 142).

### Demographic results

Table 1 shows the demographic data of the participant sample, the mean age being 57.3 years (standard deviation [SD] 12.6), with most participants being > 56 years (60.67%). Those from the postal group were significantly older than the community group (*t* = −2.09, *p* = 0.019). Females constituted 56.27% of the participant sample, but the groups did not differ in gender distribution (*p* = 0.141). There was no significant difference in the distribution of race (*p* = 0.965) and education (*p* = 0.150) between the two groups. The groups also did not significantly differ in household income (*t* = −0.72, *p* = 0.470) and period of usage (*t* = 0.79, *p* = 0.431). The distributions of the number of co-morbidities were not significantly different (*p* = 0.675).

**TABLE 1:** Demographic characteristics of respondents.

Variable	Characteristics	Community (*n* = 250)		Postal/courier (*n* = 250)	
		*n*	*%*	*n*	*%*
Gender	Male	116	46.4	48	38.4
	Female	134	53.6	77	61.6
Race†	Black people	26	10.4	15	12.0
	White people	56	22.4	29	23.2
	Mixed race people	12	4.8	6	4.8
	Asian/Indian people	155	62.0	75	60.0
	Undisclosed	1	0.4	0	0.0
Education	No formal schooling	0	0.0	0	0.0
	Primary school	5	2.0	4	3.2
	High school	107	42.8	46	36.8
	Diploma	93	37.2	41	32.8
	Degree	32	12.8	28	22.4
	Postgraduate degree	10	4.0	6	4.8
	Undisclosed	3	1.2	0	0.0
Mean age (SD) (years)	-	56.3	13.2	59.2	11.0
Period of use (SD) (years)	-	6.6	3.1	6.3	3.1
Mean household income (SD) (rands)/month	-	8211	4707	8622	5947

SD, standard deviation.

† , It is possible that the largest race group sampled are Indians as they are the second largest population in the province and are more likely than the black ethnic group to have a landline.

### Satisfaction with community pharmacy

People using community pharmacies were generally satisfied with the service they received, their mean category scores ranging from 5.30 to 7.94. The means of three of the four dimensions measured exceeded 7.6, with only financial satisfaction showing a lower mean satisfaction score of 5.3. Internal consistency of each dimension exceeded 0.52 (see Table 2).

**TABLE 2:** Satisfaction with community pharmacy.

Categories	Mean	Standard deviation	Cronbach’s alpha coefficient
General satisfaction	7.69	0.77	0.75
Technical satisfaction	7.94	0.63	0.52
Financial satisfaction	5.30	2.05	0.86
Satisfaction with counselling or explanation	7.82	0.83	0.79

### Satisfaction with postal pharmacy

Similar to community pharmacy users, participants using the postal option were generally satisfied with the service they received, with mean category scores ranging from 6.01 to 8.01 (see Table 3).

**TABLE 3:** Satisfaction with postal pharmacy.

Categories	Mean	Standard deviation	Cronbach’s alpha coefficient
General satisfaction	7.65	0.94	0.83
Technical satisfaction	8.01	0.50	0.48
Financial satisfaction	6.01	2.06	0.86
Satisfaction with counselling or explanation	7.65	0.95	0.80

### Comparison of satisfaction with pharmacy services

Category scores were compared between the groups using *t*-tests (see Table 4), with the general and technical satisfaction not differing significantly between the two groups. Participants in the postal group reported a significant difference in the level of financial satisfaction (*t* = −3.23; *p* = 0.001), while community pharmacy users reported higher satisfaction scores in counselling or explanation (*t* = 1.91; *p* = 0.028). When asked if the community pharmacy users would prefer using postal pharmacy services and vice versa (see Table 5), 6.3% of respondents in the community group and 10.4% in the postal group responded in the affirmative (*p* = 0.13).

**TABLE 4:** Comparison of satisfaction with pharmacy services.

Categories	Community		Postal		*t*-statistic	
	Mean	Standard deviation	Mean	Standard deviation	*t*	*p*
Total satisfaction	7.39	0.70	7.46	0.73	−0.89	0.374
General satisfaction	7.69	0.77	7.65	0.94	0.50	0.620
Technical satisfaction	7.94	0.63	8.01	0.50	−0.90	0.371
Financial satisfaction	5.30	2.05	6.01	2.06	−3.23	0.001†
Satisfaction with counselling or explanation	7.82	0.83	7.65	0.95	1.91	0.028‡
Adjusted satisfaction§	7.78	0.66	7.73	0.74	0.72	0.471

† , Community < Postal.

‡ , Community > Postal.

§ , Total satisfaction adjusted by removing for financial satisfaction.

**TABLE 5:** Specific patient concerns.

Specific concerns		Community		Postal		χ^2^ statistic	
		Yes	No	Yes	No	χ^2^	*p*
Alternate service	I prefer to use the alternate service to dispense my medicines.	15†	235‡	13†	112‡	2.330	0.130
Supply time	I have to wait a long time for my chronic medicines.	31†	219‡	17†	108‡	0.108	0.740
Wastage	My pharmacy dispenses medicines even though I do not need them.	10†	240‡	45†	80‡	68.182	< 0.001
Convenience	The pharmacy service I use is a convenient way to have my chronic prescriptions filled.	245‡	5†	109‡	16†	18.387	< 0.001

† , Response based on a satisfaction score ≤ 3.

‡ , Response based on a satisfaction score ≥ 4.

Specific concerns of medicine wastage, convenience of pharmacy service and time taken to supply medicines were each addressed by a single question in the questionnaire (Table 5). Community pharmacy users reported less wastage (χ^2^ = 68.182; *p* < 0.001) and better convenience (χ^2^ = 18.387; *p* < 0.001). No difference was found in the time taken to supply chronic medicines to the two groups (*p* = 0.74).

## Discussion

Patient’s satisfaction reflects the realities of care, as well as their preferences and expectations, and is often used as a marker of performance of both healthcare and healthcare systems (Larson et al. 2002). Data obtained from a patient satisfaction survey can be used to identify potential areas for healthcare services improvement, compare the quality of different care programmes and systems and detect the likely disenrollment of patients from healthcare plans (Johnson et al. 1999; Kassim et al. 2012; Panvelkar et al. 2009; Traverso et al. 2007).

In this study, a survey was conducted on patients using community and postal pharmacies to access their chronic medication in an effort to establish their satisfaction with pharmaceutical services. Postal pharmacy users are members of a medical scheme that designates postal pharmacy services as their preferred providers. The results indicate a relatively high level of satisfaction among both groups of participants. Patients using community pharmacies showed high mean satisfaction scores for three categories (> 7.69) and the lowest score for financial satisfaction, with a mean of 5.3. Postal pharmacy users showed a similar satisfaction trend, with high mean scores in three categories of > 7.65, as well as a lower score regarding financial satisfaction of 6.01. When comparing the mean total satisfaction scores of postal pharmacy and community pharmacy users, there was no significant difference between satisfaction in the two groups (*p* = 0.374). Similar findings were reported in the research conducted by Johnson et al. (1997).

Total satisfaction was then adjusted by removing the financial satisfaction scores, as this category was driven more by healthcare funder’s policy and did not give a true reflection of the pharmaceutical service the patient received. The resulting adjusted scores also showed no significant difference between satisfaction in the two groups (*p* = 0.471).

The similarity in satisfaction between the two groups was unexpected, particularly in light of anecdotal dissatisfaction with postal pharmacies (Medical Chronicle 2012, 2015; Power 2015), but is consistent with the research conducted by Johnson et al. (1997). The analysis of this difference is compounded by the heterogeneity of community pharmacies, which includes independent, chain and corporate pharmacy models in South Africa. However, this heterogeneity was not explored in this survey and could be a topic for further research. In addition, postal pharmacy is for the most part mandatory, and financial incentives are offered to patients for using postal pharmacy (Johnsrud et al. 2007; Johnson et al. 1997; Kirking et al. 1990).

Community pharmacy users reported a higher satisfaction score in the counselling or explanation category. This higher score for satisfaction was expected, as Kirking et al. (1990) explained that community pharmacists have higher potential to provide and improve patient medication knowledge and thereby enhance compliance. Community pharmacists can also elicit additional concerns during face-to-face consultations and provide a more personalised service (Larson et al. 2002; MacKeigan & Larson 1989).

The South African private healthcare sector has seen some healthcare funders and PBMs move to the mandatory use of postal pharmacy to control medicine costs. This model is also being used by the public sector, with the introduction of the integrated chronic model (Department of Health 2013; Naidoo 2013). This practice has been adopted despite the poor evidence that healthcare funders derive a cost benefit when community pharmacies are substituted by a postal option. Researchers in the United States reported that medical funders paid a higher medicine cost when using postal pharmacies (Carroll 2014; Carroll et al. 2005; Johnsrud et al. 2007).

Valluri et al. (2007) determined that postal pharmacy services increase drug product cost if the cost per unit is not reduced. This does bring into question the mechanisms used by postal pharmacies in South Africa to reduce costs, specifically in light of medicine pricing regulation that promulgates single exit price (SEP) for all medicines in the country. A media article (Medical Chronicle 2012) published in 2012 explained this higher cost as postal pharmacies charging a composite fee per item, which includes non-healthcare costs not charged by community pharmacy, such as administrative call centre, and extensive data analysis costs. In addition to the cost of the medicine, healthcare funders have not adequately addressed anecdotal concerns of both patients and pharmacists relating to freedom to choose pharmacy providers and thereby address issues of product integrity, wastage, convenience and supply continuity (Kirking et al. 1990; Rupp 2013).

The postal group showed a higher score for financial satisfaction, which is consistent with healthcare funders encouraging the use of postal pharmacy with an economic incentive (no co-payments). The legality of high co-payments has been questioned, as patients using a non-designated service provider accrue co-payments of between 20% and 40% of the total medicine cost (Medical Chronicle 2012, 2015). Similar concerns were raised by Clark et al. (2009), where self-dealing practices by PBMs that function both as processors and postal pharmacy vendors have pointed to the use of incentives to modify patients’ behaviours in ways that may benefit them, the postal pharmacy and pharmaceutical manufacturers.

It is interesting to note that financial satisfaction had the lowest satisfaction score in both population groups, which was also reported by Johnson et al. (1997). Thus, both groups reported that they do not think they pay too much of a co-payment or their medical scheme does not pay enough towards medicines. When comparing prescription benefit plans between community and postal pharmacy, Clark et al. (2009) revealed that the absence of a co-payment incentive to use a postal pharmacy was associated with its lower use and costs to medical schemes.

The choice of patients to remain with postal pharmacies was also unforeseen but can perhaps be explained by their mandatory use and the incentives offered by healthcare funders to encourage their use. Rupp (2013), who studies attitudes towards postal pharmacy, concluded that patients oppose any restrictions on their freedom to use the pharmacy of their choice as a matter of general principle and that this opposition was evidenced even among patients who were generally satisfied with postal pharmacies. Desselle (2001) also confirmed that satisfaction decreased if patients perceived the funder and pharmacy network to be overly restrictive, with this opposition being evident even among patrons who were generally satisfied with postal pharmacies (Rupp 2013).

Although community pharmacies have long claimed to be superior in being able to supply medicines to patients promptly, there was no significant difference between the two groups. The comparison of medicine delivery systems between the two groups was affected by the range of factors affecting medicine supply, many of which have not been quantified (Kirking et al. 1990) and are currently based on anecdotal evidence (Magubane 2014). Since late 2014, a number of extended postal services strikes in South Africa have affected the distribution of chronic medicines. This led to media reports of patients’ dissatisfaction as they were not able to get their chronic medicines (Magubane 2014; Times LIVE 2014). Our research did not reflect the impact of the postal strike and its ensuing patient dissatisfaction.

The postal pharmacy group reported a significantly higher level of wastage, with 36% stating that they received medicines they did not need. Medicine wastage was reported in previous studies comparing the costs of community and postal pharmacy (Kirking et al. 1990). Researchers in the USA detailed medicine wastage at 15% when using postal pharmacy, with an overall 4.8% cost increase to the medical funder (Carroll et al. 2005).

Kamei et al. (2001), in their research in Japan, reported that communication and convenience were the functions that patients most desire in their pharmacy. The claim made by community pharmacists in South Africa, that patients find using community pharmacy more accessible and convenient (Magubane 2014), was reflected in our research. Community pharmacy users reported a higher level for convenience when compared to those using the postal option.

## Conclusions, limitations and recommendations for future research

This study showed that there was no significant difference between the satisfaction scores of patients utilising community or postal pharmaceutical services to access their chronic medicines. However, measuring satisfaction within the two categories may be useful to continually improve pharmaceutical services by both the pharmacy and healthcare funders.

Funders who advocate or are considering advocating the use of postal pharmacy should consider their beneficiaries’ needs regarding choice and convenience when negotiating contracts with pharmacy providers, which might exclude some pharmacies from participating in the network. Collaboration between healthcare funders and community pharmacies could result in the development of pharmaceutical care programmes that stress the quality of care, standardised services and professional compensation for pharmacists, but this needs to be investigated further. This type of collaboration may result in a more satisfied and involved patient and ultimately result in better health outcomes and lower healthcare costs, but a national study needs to be conducted.

A limitation of this study is that it did not include different pharmacy types within the community pharmacy group, as patient satisfaction could have been affected by patronage of the different pharmacy types, that being corporate, retail and independent pharmacies. In addition, the sample population was limited to the eThekwini municipality and the largest population group represented was Indians, which is the second largest population group in eThekwini and possibly the largest race group owning a landline telephone. The findings are thus limited to those with telephone landlines.

Although most results were consistent with the literature, additional research is necessary to determine if these results can be extrapolated to the general population of South Africa. Future investigations also should consider the impact of the different pharmacy types within the community pharmacy group on patient satisfaction and availability of pharmacies in the different districts of South Africa. Finally, with the move to National Health Insurance, policymakers need to ensure that they provide high-quality pharmaceutical services and are more inclusive of community pharmacies to deliver quality care.
